# A Survey of Anomaly Detection in Industrial Wireless Sensor Networks with Critical Water System Infrastructure as a Case Study

**DOI:** 10.3390/s18082491

**Published:** 2018-08-01

**Authors:** Daniel Ramotsoela, Adnan Abu-Mahfouz, Gerhard Hancke

**Affiliations:** 1 Department of Electrical, Electronic and Computer Engineering, University of Pretoria, Pretoria 0002, South Africa; a.abumahfouz@ieee.org (A.A.-M.); ghancke@ieee.org (G.H.); 2 Council for Scientific and Industrial Research (CSIR), Pretoria 0184, South Africa; 3 Department of Computer Science, City University of Hong Kong, Hong Kong, China

**Keywords:** industrial informatics, industrial sensor network, cyber-physical systems, critical infrastructure, water monitoring

## Abstract

The increased use of Industrial Wireless Sensor Networks (IWSN) in a variety of different applications, including those that involve critical infrastructure, has meant that adequately protecting these systems has become a necessity. These cyber-physical systems improve the monitoring and control features of these systems but also introduce several security challenges. Intrusion detection is a convenient second line of defence in case of the failure of normal network security protocols. Anomaly detection is a branch of intrusion detection that is resource friendly and provides broader detection generality making it ideal for IWSN applications. These schemes can be used to detect abnormal changes in the environment where IWSNs are deployed. This paper presents a literature survey of the work done in the field in recent years focusing primarily on machine learning techniques. Major research gaps regarding the practical feasibility of these schemes are also identified from surveyed work and critical water infrastructure is discussed as a use case.

## 1. Introduction

Industrial wireless sensor networks (IWSN) have gained popularity in recent years and are being used in a variety of different applications [1,2,3,4,5]. One of their most important applications involves critical infrastructure, where they form part of supervisory control and data acquisition (SCADA) systems [6]. They are popular in these systems because they provided the same control features as their wired counterparts but have a much lower deployment and maintenance cost. These systems also introduce the potential for intelligent monitoring and control within the Internet of Things (IoT) application environment. The rapid rise of IoT technologies has meant that in the future these devices are going to be an integral part of smart city applications. A recent study has shown that by 2020 there could be over 100 billion IoT applications in existence and the market value for these projects is currently over 11 billion Euro in Europe alone [7]. The complex nature of the application environment also means that IWSNs require a multidisciplinary team of experts for both development and application [8]. In the application domain these networks are usually deployed in hostile environments and their resource constraints introduce several security challenges [9,10,11,12]. This is particularly problematic in these critical infrastructure applications because compromised systems can lead to disastrous consequences for the underlying infrastructure. In this scenario control theory and network security are combined to protect the system and this is collectively referred to as cyber-physical security.

These cyber-physical systems [13] can be conveniently monitored and controlled remotely but this added convenience comes at the cost of increased vulnerability. This is because attackers can compromise the corporate network using conventional network security attacks and use this access to seize control of the SCADA system. This was the case in the attack on the Ukrainian power grid [14] which resulted in over 200,000 consumers not having access to electricity due to power outages. Intrusion detection provides a second layer of security in case of the failure of normal network security policies and the system becomes compromised. This secondary defence is paramount in these applications because it would limit the resultant damage of a compromised system. An example of this can be seen from the Maroochy water treatment system attack where it took three months to detect that the system was compromised and during that period 150 million litres of untreated sewerage was released into local waterways [15].

An important consideration of protecting IWSNs is that conventional network security policies cannot be applied to these networks in their original format because they are deployed in vastly different environments and thus have conflicting priorities to conventional IT networks. This is especially the case with intrusion detection schemes which are conventionally resource intensive [16]. Anomaly detection is a branch of intrusion detection which is popular in IWSNs because of its flexibility and relative resource friendliness [17]. An anomaly is defined as something which deviates from the norm/standard. In the application environment, an anomaly can be found by analysing the sensed data or traffic patterns to find abnormal behaviour in the system. The sensor nodes in IWSNs consume the most energy while transmitting data as opposed to the data processing tasks [18,19,20,21]. A major limitation in these systems is the finite power supply which is usually a conventional battery. It is for this reason that anomaly detection algorithms in this application scenario should seek to limit the amount of communication required to perform the task [22]. This can be achieved by making use of distributed in-network processing.

Another important consideration is that an anomaly might not necessarily be the result of a security breach [17]. A faulty node could produce abnormal results which could intern bring down the entire network. In critical infrastructure applications these types of anomalies are as dangerous as those caused by intruders and should also be detected by proposed schemes. Several anomaly detection surveys which give comprehensive overviews of the relevant literature exist [23,24,25,26]. These surveys however do not specifically consider resource constrained IWSN, which have conflicting priorities to conventional computer networks. To the best of our knowledge, there is also no survey which considers the applicability of anomaly detection to critical infrastructure applications (specifically water systems) taking the above into consideration. Intrusion detection systems specifically designed for WSNs are surveyed by the authors in [16,27]. These schemes are however distinguishable from their anomaly detection counterparts which provide greater flexibility in these dynamic environments. The authors in [17,28] surveyed the state of the art anomaly detection schemes with the former presenting a guideline for the selection of appropriate schemes depending on the specific application requirements. A brief review of anomaly detection in traditional SCADA systems was presented by the authors in [29] but this did not specifically consider IWSNs. This paper presents a brief overview of the work done in the field in recent years and highlights the practical considerations of the proposed schemes in these resource constrained environments. Critical water system infrastructure was chosen as the primary application of concern because of the increased number of cyber-attacks against water utilities in recent years [30]. The importance of protecting these systems is paramount and this paper looks at the challenges and limitations of this within the broader IWSNs anomaly detection application environment.

Anomaly detection schemes have been classified based on different indicators (Section 2), for example, based on the detection model they can be classified into parametric (Section 3) and non-parametric (Section 4) models. This paper focuses more on the latter and investigates several non-parametric schemes which are more suitable for IWSNs because of their dynamic nature. Critical water system infrastructure is used as a case study to examine the applicability of these schemes to SCADA systems (Section 5) and some applications challenges are then discussed (Section 6). The schemes are then compared using several different performance measures as well as the inherent challenges and limitations (Section 7). Some open issues for future work are also discussed in the paper. This paper highlights important issues that need to be addressed in order for the field to evolve from being primarily concept-based, to one that is practically viable.

## 2. Anomaly Detection

Abnormal changes within monitored IWSN systems can be detected using anomaly detection algorithms [31]. In cyber-physical systems for instance, a fault in system the system caused by general wear and tear would cause significant changes to the sensed data which could have disastrous consequences. The same is true if an intruder were to successfully feed false data into the system in an attempt to affect the system functionality. Both of the examples would result in data that deviates from norm and should be flagged by anomaly detection.

**Figure 1:**
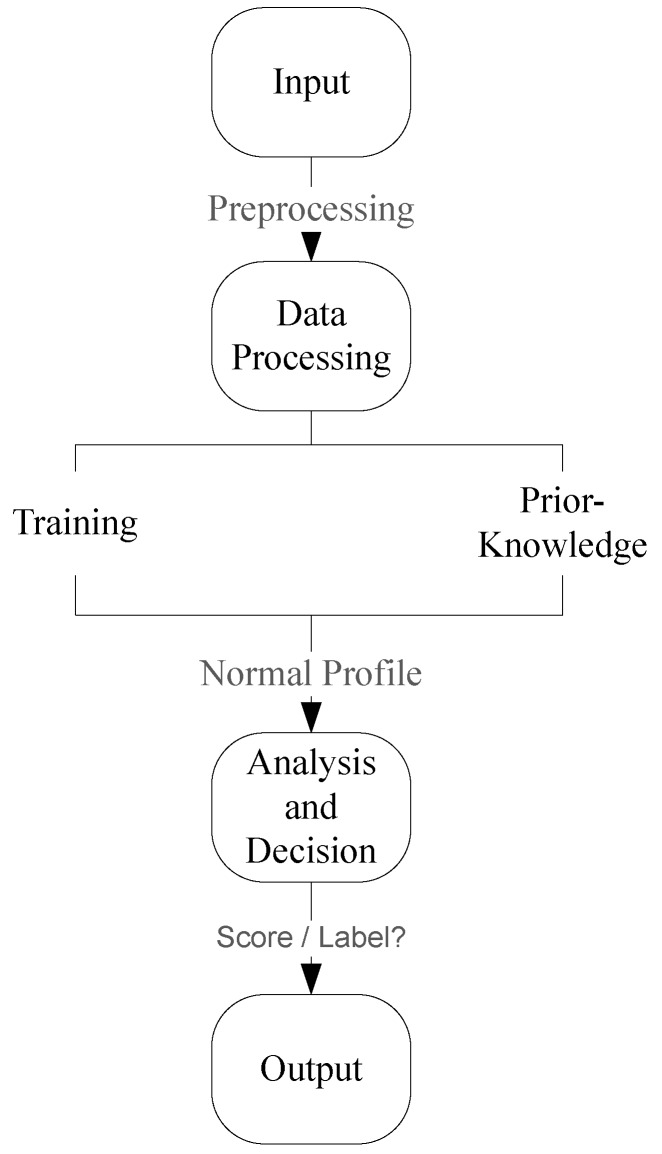
Generic Anomaly detection framework.

Figure 1 shows a generic framework for anomaly detection [32] which was derived from the generic intrusion detection framework. The input to an anomaly detection scheme is a dataset which consists of data instances which in turn consist of data attributes. Depending on the detection scheme, the data may need to be pre-processed before proceeding further. A normal profile is then produced through data processing by using either a training procedure or prior knowledge. Once a normal profile is produced, a test instance can be classified as anomalous with the aid of some established threshold.

Anomaly detection schemes can be categorised using several different indicators. The model used in detection schemes is a popular indicator used to classify them. The two general categories in this regard are parametric (also known as statistical) and non-parametric models [28]. Parametric methods are suitable in stable environments where the data distribution is well known and unlikely to change frequently. Non-parametric models can be used in dynamic environments where the statistical distribution is unknown and unfamiliar. The first category provides faster detection while the second category provides detection generality. Table 1 shows a comparison between parametric and non-parametric schemes.

**Figure 2:**
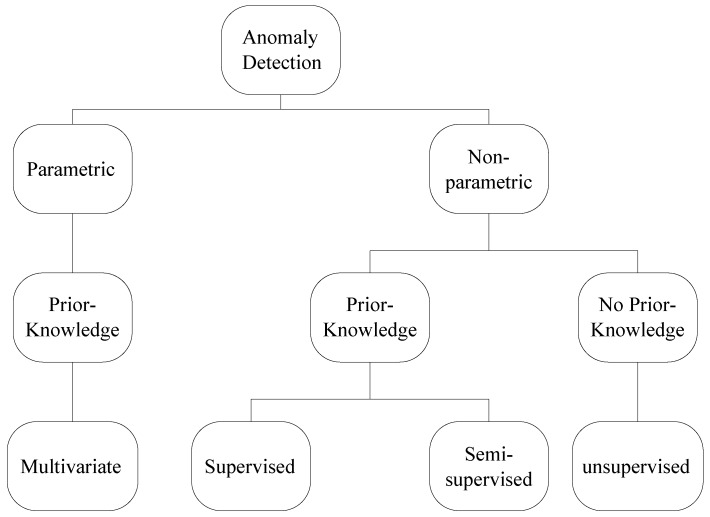
Anomaly Detection Summary.

Anomaly detection schemes can also be broadly classified as those that require prior knowledge of the system data and those that do not [23]. As with the previous categorisation, the first category provides faster detection while the second category provides detection generality. In the first category the classifier can either use supervised or semi-supervised learning [33]. The former uses a large training set and has to be retrained if normal system behaviour changes while the latter generalises results using a smaller data set and adapts to changing system behaviour on the fly. The second category, which does not require prior knowledge, uses unsupervised learning and assumes that abnormalities are far removed from the norm. Table 2 shows a comparison between the different training methods and this section is summarised in Figure 2. This paper focuses on these two categorisation because they focus on information designers need to have about the data before they can use the algorithm which, as will be explained later, is paramount in anomaly detection schemes.

## 3. Parametric Methods

Parametric methods require prior knowledge of the density distribution of the data being analysed [28]. An anomaly is assumed when a data point that has a small probability of occurring is observed. Parametric methods can use either univariate or multivariate distribution functions with the latter being more suitable for IWSN applications. The former only deals with one random variable at a time so each variable would have to be modelled independently using its own distribution function while the latter allows vectors of random variables to be modelled using the same distribution function. Parametric methods are suitable when the nodes and the distribution function will be static over the network lifetime.

### Multivariate Distribution Functions

A scheme that detects sinkhole and Denial of Service (DoS) attacks is proposed by the authors in [34]. This scheme detects global long term anomalies that cannot be detected by conventional point-based techniques. As with most IWSN applications, a decentralised approach is followed in order to decrease the communication overhead. The scheme uses a Kullback-Leibler divergence-based algorithm that employs segment-based recursive kernel density estimation. It was found to have generally favourable results although dimensionality is still a major issue.

The problem of data loss/modification was addressed by the authors in [35] who developed a scheme that can both detect and recover lost data. Both detection and recovery use a partial least squares and principal component-based multivariate technique but the latter additionally utilises trimmed scores regression. Both models had favourable results with the latter performing best when there is a strong correlation between the data of the compromised node and its neighbours. In both cases, the selected routing algorithm affected both the anomaly detection and the effect the compromised data has on the system.

The aforementioned problem can be alleviated by using a dynamic multi-hop routing strategy [36]. In static routing, data always travels via the same route to the sink while in dynamic routing the next hop is chosen using a random selection process. In this way, the effect of the compromised data is spread across the neighbourhood making it easier to manage. While this technique improves the data recovery, a major drawback is that it introduced a traffic imbalance which can affect the energy consumption of the nodes. In addition to this, it becomes more difficult to find the source of the attack because its effects have been spread over a larger area.

## 4. Non-Parametric Methods

When the density distribution of the underlying data is not known in advance, then non-parametric methods can be used for anomaly detection [28]. These methods are ideal for resource constraint devices in dynamic settings. This can be the case when the monitored environment and/or the network topology are likely to change over time. There are numerous methods that have been proposed in this category [37,38,39,40] but this paper will focus on methods that employ computational intelligence. More specifically, pattern recognition through machine learning where a known dataset is used to find the input/output relationship of the system which can in turn be used to classify an unknown dataset.

### 4.1. K-Nearest Neighbour

K-nearest neighbours (kNN) anomaly detection schemes are generally classified into two broad categories, those that are density-based and those that are distance-based [41]. The former is ideal for multilevel detection applications and can process unevenly dense training data. Two popular metric parameters in these schemes are the local outlier factor (LOF) and the connectivity-based outlier factor (COF) which both have large computational complexities that may not make them feasible for IWSN applications without some optimisation. Distance based schemes classify a data point as anomalous to a dataset if less than a predetermined number of points in that set are a predetermined distance from that set. These schemes also have high computational complexities but can be optimised for IWSN applications.

A density-based kNN scheme that uses LOF as a metric for anomaly detection on uncertain data was proposed by the authors in [42]. Local density and uncertainty information is used to calculate uncertain local outlier factors (ULOF) in an uncertain dataset. The distance distribution function is found by using the least squares algorithm on multi-times curve fitting and a pruning algorithm is used to reduce the set of potential “nearest-neighbours”. Experiments yielded favourable results when compared to state-of-the-art techniques, but dimensionality was found to be a major drawback.

The lazy-learning problem of the kNN algorithm was addressed by the authors in [41] who proposed a distance-based scheme that makes online detection a possibility. The scheme is based on a hypergrid that consists of several fixed-size hypercubes in which data points are mapped in order to make an anomaly detectable. Hypergrid schemes have low computational complexities making them ideal for IWSN applications. In fact, the complexity of the proposed scheme increases logarithmically with sample size as opposed to other schemes that have quadratic increase.

The effectiveness of a kNN algorithm lies with the optimal selection of the k-value. A larger k-value makes the algorithm less susceptible to noise but could also result in a neighbourhood that is too large with indistinct boundaries and cause short circuit errors. The opposite could inadvertently lead to data points in the same class being separated due the weakened association between the nodes in the same neighbourhood. In [43] the authors propose a weighted natural neighbour graph (WNaNG) which is an adaptive algorithm that does not make use of the k-value. The algorithm makes use of the principle of implicit trust to find all the “friendly” nodes in a particular cluster. The rest of the nodes are then considered “outsiders”. The algorithm has generally favourable results when compared traditional kNN algorithms depending on the data distribution and selected k-value.

### 4.2. Support Vector Machines

Support Vector machines (SVMs) are traditionally supervised learning classifiers that require labelled training data to map samples into one of two classes [44]. These types of SVM models are called binary or two-class linear models. Binary SVMs can be adapted to work with non-linear data by mapping them into a high dimensional feature space using the Kernel method. Structural risk minimisation (SRM) is achieved when a maximum margin hyperplane can be found. In cases where unsupervised learning is required, which is normally the case in anomaly detection schemes because of the lack of training data, one-class SVMs can be used. In one-class SVMs the only class is the “normal” class and anything which deviates from the norm is classified as an anomaly.

The use of a sliding window-based Least Squares SVM for online outlier detection was proposed by the authors in [45]. The algorithm uses a Reproducing Kernel Hilbert Space (RKHS) with Radial Basis Function (RBF) kernel. This kernel requires the system that produces the readings to be in steady state otherwise the detection performance deteriorates. The proposed algorithm addresses this issue by using the norms of the differences between samples and Least Squares Estimates instead of Euclidean norm. The modified scheme was shown to significantly outperform the original but is much slower.

An anomaly detection scheme based on SVM and Linear regression models is proposed for medical applications by the authors in [46]. The SVM detects anomalies and the linear regression model is used to find the abnormal sensor measurements. A patient’s vital signs can sometimes deteriorate suddenly and seemingly without warning but this model is able to distinguish between those cases and erroneous sensor readings. The system was tested using real patient data and was found to have a higher true positive and lower false negative rate than state-of-the-art techniques.

As already mentioned the lack of training data is a serious drawback of binary SVMs. The same is true for multi-class SVMs because they are an extension binary SVMs that allow for sample categorisation into multiple classes. Moreover, should the training data be available SRM remains a major issue in these schemes and as such new emerging anomalies are difficult to detect. A multi-class SVM for anomaly detection in large scale deployments that seeks to address this issue is proposed in [47]. It is capable of classifying data into multiple known classes and uses the unknown class to detect anomalous data. The scheme is an extension conic segmentation SVM (CS-SVM) which is known as a true multi-class SVM as it does not use a combination of binary SVMs to achieve multi-class classification. The added functionality of the CS-SVM is achieved by combining it with the one-class SVM. The proposed scheme was tested using several benchmarking datasets and had favourable results.

### 4.3. Artificial Neural Networks

Artificial Neural Networks (ANNs) model the way the biological brain solves problems to enable computational systems to have the same capacity to learn [48]. They consist of several interconnected artificial neurons that are categorised into 3 main layers: the input, hidden and output layers. The ability of neural networks to detect patterns in the input/output relationships of given data sets makes them ideal contenders for anomaly detection systems. However, their inability to handle unseen data is a major limitation for these kinds of applications so ANNs are seldom used in isolation [49].

An adaptive neural swarm approach was proposed for anomaly detection by the author in [50]. The system uses a decentralised collaborative approach which utilises agent-based swarm intelligence. Modular neural networks are formed autonomously by the swarm agents and the reinforced learning approach is used in the training process to facilitate an unsupervised learning environment. The proposed scheme is theoretically capable of monitoring the application environment and identifying possible anomalies in real-time. Simulation results were promising, but more research is required to explore the feasibility in resource constrained devices.

An interesting research focus in the application of neural networks in anomaly detection is on the training process. A variation of the online sequential extreme learning machine (OS-ELM) was proposed for the training of single layer feed-forward neural networks (SLFN) by the author in [51]. The proposed scheme uses an embedded fixed-point implementation which has overcome the stability issues inherent with fixed-point implementations. The scheme is ideal for resource constrained devices because the underlying algorithm has a low computational overhead. When compared to state of the art techniques, it was found to have comparable results.

Another related application scenario is the deployment of IWSNs for the specific task of detecting anomalies. In this case, the anomalies are usually caused by equipment malfunction but as already mentioned, there is little difference between the effects faulty equipment and an attack. An architecture for industrial condition monitoring that is based on a dynamic neural network was proposed by the authors in [52]. The proposed scheme was designed to work in noisy, dynamic non-linear systems and was tested on the cooling system of a poly-phase induction motor. It was able to produce a high detection accuracy when tested using unseen anomalous data.

### 4.4. Genetic Algorithms

Genetic algorithms (GA) are based on the principle of natural selection. Given a population of possible solutions, the GA uses a subset of them (referred to as parents) to create the “next generation” which will also be used to create their successors and so on. This evolutionary process continues until it produces an optimal solution. The different types of GA’s differ in the way “parents” are selected and how the “next generation” is produced.

A GA-based node attribute behaviour anomaly detection scheme was proposed by the authors in [53]. The scheme uses the fact that particular node attributes can be used determine whether or not a particular node is behaving “normally”. These attributes can then be mapped into the feature set which is used by the GA to detect anomalies. The proposed scheme is capable of adapting to changes in system behaviour over time. The system was simulated in NS-2 and yielded a detection accuracy of 87.5% with a false positive rate of 12.5%. The authors however noted that these values can be significantly improved by using more behaviour indices.

A GA based scheme that be used to improve reputation systems was developed by the authors in [54]. The scheme uses trust and reputation principles to identify misbehaving nodes and then isolate them from the network. The reputation of a node is determined by an unsupervised GA which monitors the network for suspicious behaviour. Nodes that are misbehaving will have their reputation scores sharply reduced until it is deemed to be untrustworthy. Should the misbehaving node start to behave “normally” its reputation score will gradually increase until it becomes trusted again. This possibility for redemption accounts for selective behaviour of nodes, which may not be the result of an attack. The proposed scheme had a high detection accuracy and low false positive rate when tested with known attacks.

Besides GAs, there are other biologically inspired artificial intelligence algorithms that can be used in anomaly detection schemes. For instance, the use of the negative selection algorithm (NSA) of the artificial immune system (AIS) to detect anomalies was proposed by the authors in [55]. The AIS is modelled after the human immune system and is advantageous because it is inherently adaptive making it ideal for anomaly detection schemes. The proposed scheme uses a modified version of NSA (modelled after the natural system of the same name) that has an added injection feature step referred to as vaccination. This allows for an ad hoc update of the detector set without having to retrain the system. Experimental results show that the proposed scheme outperforms state of the art techniques

### 4.5. Hybrid Systems

In intrusion detection systems, the traditional definition for a hybrid system is one that combines signature-based classifiers with anomaly detection methods [24]. In this paper that definition will be extended to include all systems that combine two methods to produce a stronger intrusion detection system. While increasing in popularity, hybrid systems do not always result in a better overall system because what you gain in detection accuracy could be lost in efficiency. So the challenge in these kinds of systems is creating robust schemes that have high detection accuracies and low false positive rates while still being feasible for practical implementation.

The use of a hybrid model that combines signature-based classification and anomaly detection using SVM was proposed for intrusion detection by the authors in [56]. The system is designed to work for networks that use clustered architectures where cluster heads are elected to forward aggregated data packets to the base station. The cluster heads are not more powerful than the other sensor nodes and are selected based on the remaining energy of the nodes in the cluster. Each node runs a local intrusion detection agent while the cluster head runs the global one which is responsible for making decisions based on two detection schemes. The proposed scheme yielded a high detection accuracy and low false positive rate when tested with a range of known attacks. It also had better or comparable result to state of the art techniques.

A scheme that uses a combination of spectral clustering (SC) and deep neural networks (DNNs) for intrusion detection was proposed in [57]. SC is used to improve the performance of DNNs which usually perform poorly when exposed to camouflaged attacks. In the learning phase, spectral clustering is used to extract features and divide the data into categories depending on their similarities using cluster centres. Each category is then used to train a DNN which then learns specific characteristics of each category. Once trained, the DNNs detect intrusions by first categorising the input data into the above mentioned categories using SC. Based on the learned behaviour, the DNNs are able to distinguish between normal and malicious behaviour. The proposed scheme outperforms state-of-the-art techniques when tested using known datasets. The scheme was tested using the simulator NS-2. However, no evaluation was conducted to verify the feasibility for practical implementation given the scarce resources in the application scenario.

One could go further than hybrid systems by using an ensemble of algorithms to detect anomalies. This would theoretically result in a better detection accuracies than hybrid systems because there are more algorithms working together to overcome each other’s limitations. Owing to the resource constraints of IWSNs, these types of systems are not as popular in these applications. The authors in [58] however proposed a framework for the use of ensembles of incremental online learners for anomaly detection. Their decentralised approach was able to outperform their centralised offline learner counterparts. While the complexity of the algorithms used will ultimately be the determining factor, they find that ensemble schemes are feasible for practical implementation.

## 5. Anomaly Detection in Water Systems

In March 2000, the water treatment system of Maroochy in Queensland Australia was compromised by a disgruntled attacker who was turned down for a job at the facility [15]. The attacker ceased control of 150 pump stations remotely and in the three months it took to detect the breach he was able to release 150 million litres of untreated sewage into local water ways. The system did have security mechanisms in place but the attacker was involved in the installation of the upgrades to the system and used this knowledge to bypass them. The system exhibited unexplained behaviour during this period which was eventually noticed by the engineers who were eventually able to track him down and have him arrested. At this point however, the attacker had already caused a substantial amount of damage. This example will be used to illustrate the benefits anomaly detection in critical infrastructure applications by looking at how the system failed and how anomaly detection could have been used to limit the damage.

Some of the most notable faults exhibited by the system during the breach were [59]: (1) unexplained alarms, (2) increased network traffic, (3) misbehaving pumps, (4) alarms not going off when they were supposed to, and (5) communication lockups. These faults can clearly be classified as system anomalies which can be classified by detection schemes. SCADA systems consist of the corporative and the control networks [6]. The former is responsible the general system supervision while the latter is more intimately involved with the control of the various subsystems. This discussion will be constrained to the control network which is where the sensor networks are located. Each of the notable system faults will now be discussed to ascertain how an anomaly detection scheme at the control level could have led to the breach being detected sooner.

The system exhibited several alarms which could not be explained by the administrators. Anomaly detection schemes however deal with raw system data at the control level. This means that a detected anomaly can be isolated to a specific part of the network which would have made it easier find the source of the problem. In this instance, the administrators would have been able to approach the problem from the sensor level, e.g., specific sensors within the system are measuring uncharacteristic values based on historic data. The problem of alarms not going off when they were supposed to could have also been aided by these schemes. In this case however, this would have only been useful once the problem had been detected. The alarms still wouldn’t have gone off because that was modified at an administrative level, but the anomalies would have been detected and logged making it easier to find the source. The other three faults were general anomalies which would have been easily detected by any anomaly detection scheme.

The attack on the Maroochy water system was however not very sophisticated which is why it could have been detected easily using appropriate schemes. The authors in [60] used a water treatment dataset to show that more sophisticated attacks can also be detected in water systems. In this case the system was tested against a variety of spoofing attacks which could lead to, among other things, overflow and underflow errors. The former can lead to flooding and the latter can potentially damage the pumps. The system was able to detect all the various attacks it was tested against and isolate the anomalies to specific sensors within the system. This would make it easier for a system to recover once compromised because administrators would know the exact location of the problems.

**Figure 3:**
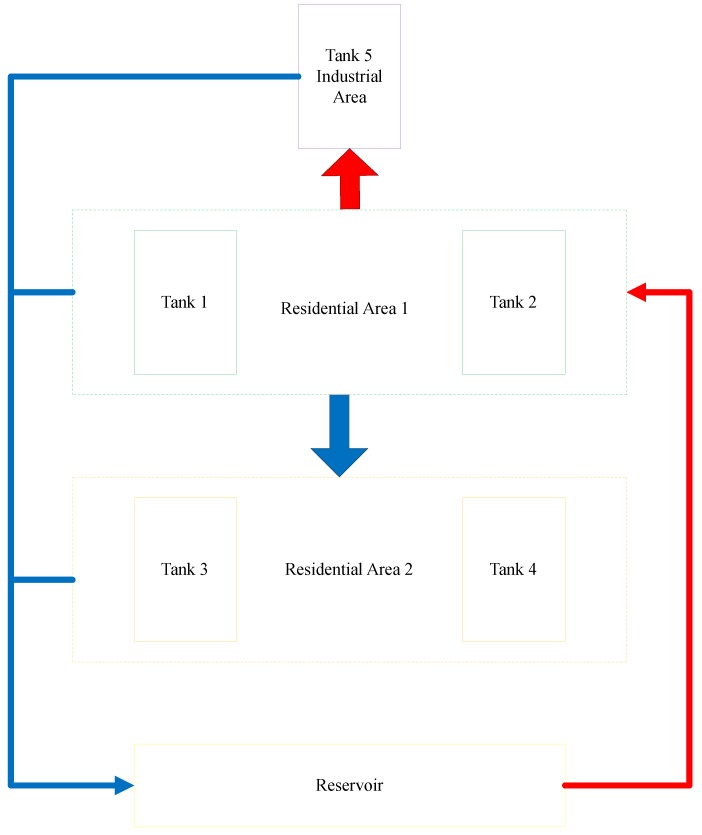
FACIES Testbed.

The authors in [61] went further by using the FACIES testbed to compare model-based fault detection (FD) and network anomaly detection. The FACIES testbed emulates the water system of the fictional HighLake City which primarily uses gravity to supply water to the different areas of the city as shown in Figure 3. The testbed consists of 5 tanks which represent different areas of the fictional city and a reservoir tank which serves as the main water source and sink. The reservoir supplies water to residential area 1 through tanks 1 and 2 using pumps. Residential area 1 supplies water to the industrial area (through tank 5) using a pump and also to residential area 2 (through tanks 3 and 4) using gravity. The configuration allows several different water distribution scenarios to be emulated and has many predefined fault mechanisms that can be used to test and evaluate algorithms in the application scenario. The authors in [61] used this testbed in their evaluation and found that FD is not an adequate mechanism to detect attacks because an attacker with sufficient knowledge of how the system operates would easily be able to shield their activities from detection. They however found that anomaly detection still works even when the attacker attempts to go unnoticed by launching stealthy attacks. The introduction of anomaly detection also makes it possible to distinguish between physical faults and attacks.

It is thus evident that anomaly detection is a convenient second line of defence in critical water system applications. It is however necessary to discuss the applicability of these schemes taking two critical points into consideration. The first important consideration is that of prior knowledge, which in this and most other existing critical infrastructure applications is not a major issue when considering physical faults. Labelled training data is it pertains to system attacks is however not as easily available making this a major limiting factor. The process dynamics of real world critical water infrastructure will evolve over time meaning that proposed anomaly detection schemes should take this into consideration [62]. The use of simulated data is useful in determining the viability of the proposed schemes but may not adequately portray the challenges of practically deployed full-scale system. This is especially true when considering the fact that anomalies are rare obscure events in practice which creates additional challenges as opposed to the simulated scenario where they are artificially created and clearly defined. An example of this can be seen in [63] where the authors used the real world Vitens Innovation Playground dataset which they found to lack sufficient logged information about anomalies. This lack of labelled training data severely hindered the scheme’s performance because there was not enough information to enable them to adequately train the algorithm.

It may not always be possible to obtain real world training data for full-scale systems especially in the case of system attacks which are even less frequent than system faults. It is however necessary for proposed schemes to be trained and tested against a variety of such attacks but as mentioned before simulated data may not always be adequate. This is where scaled down practical systems such as the FACIES testbed mentioned earlier could be useful. These scaled down versions of real world systems enable designers to work with system data as close to practically deployed systems as possible. The use of such systems could greatly improve accuracies because the full impact of modelled attacks can be observed and designers would be able to make informed decisions about aspects such as sensor placements and what the appropriate action should be taken when an anomaly is detected [64]. The latter is important because some anomalies could have devastating consequences if corrective action is not implemented in a timely manner, even just as an interim measure while the administrator investigates.

The computational overhead of the detection schemes is also another limiting factor in terms of their applicability in practice. Proposed schemes should run in parallel with the control algorithms without significantly affecting their performance. This is particularly important when considering the bedrock of network security, the CIA triad, which identifies the three key components that need to be protected in most systems: confidentiality, integrity and availability. In SCADA systems, availability has been identified as the most important of these three components [65]. Availability is more difficult to classify than the other two components because its protection cannot be evaluated using a binary measure. A system can still be able to carry out its normal operations but be slowed down enough to affect its overall performance making it impossible to carry out its mandate. In this case an anomaly detection scheme which significantly affects system performance will be counterproductive because it will have affected the availability of system resources to the point of constituting an anomaly or breach. The two considerations mentioned above are paramount in order for these schemes to be seriously considered in critical infrastructure applications.

The final part of this discussion considers the fact that traditional SCADA systems are protected using physical FD mechanisms and are never directly connected to the Internet presumably making them safe from remote attacks. This may lead designers to believe that not upgrading the system to take advantage all the benefits that come with smart interconnected devices may be a small price to pay to secure it. In this scenario the belief is that the FD mechanism would be adequate protection for the system because the system would then be safe from cyber-attacks. This belief would be false as was demonstrated by the Stutnex worm attack of 2010 [66]. The worm was specifically designed to target computers that were running Siemens Step 7 which is used by many SCADA systems. Once active the worm would directly attempt to communicate with PLC (programmable logic controller) devices within the system. The most troublesome aspect of the worm was that its propagation mechanism did not require an internet connection to spread which made it capable of compromising legacy systems as well. This demonstrated the fact that traditional SCADA systems are not inherently safe from cyber-physical attacks.

## 6. Applying Anomaly Detection in Water Systems

In Section 5, anomaly detection schemes in critical water system infrastructure were broadly discussed and some practical challenges were outlined. Anomaly detection was then introduced in more detail in the following sections and some state of the art techniques were discussed. In this section, anomaly detection in the application scenario will be discussed by making use of a security attack to illustrate what can go wrong and then using that to discuss some practical considerations to take into account when applying anomaly detection from a machine learning perspective.

### 6.1. Data Integrity Attack

**Figure 4:**
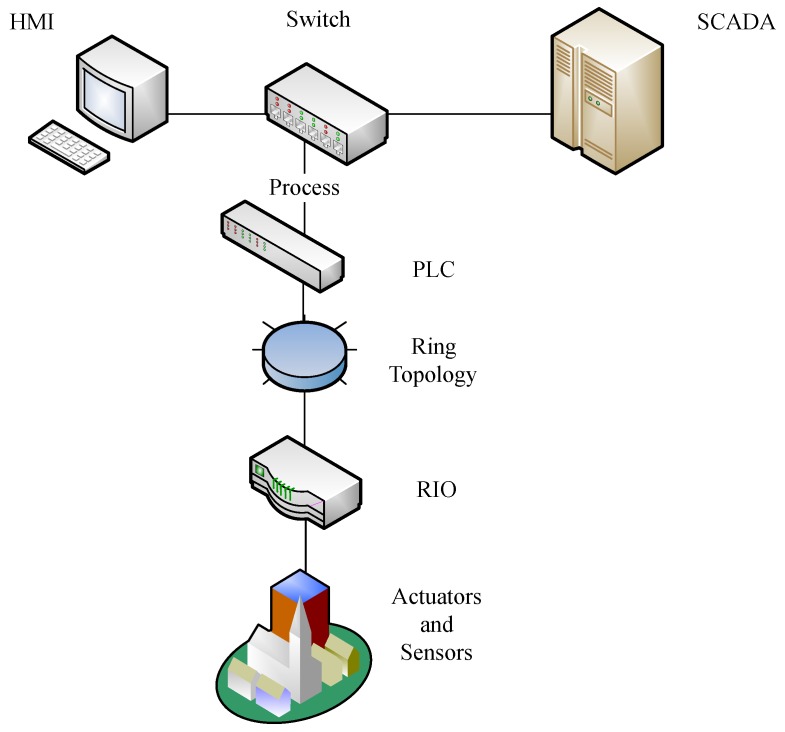
SWaT Industrial Control Network.

The authors in [67] attack the fieldbus communications of the Secure Water Treatment (SWaT) testbed using a Man-in-the-middle (MitM) attack to illustrate the devastating consequences such attacks could have in industrial control systems (ICS). The SWaT testbed is a scaled down version of a water treatment plant that has six main processes which emulate the functionality their full-scale counterparts. The industrial control network for the SWAT testbed is shown in Figure 4. In the network, the SCADA system communicates with the PLC which in turn has a ring topology connection with the RIO (remote input/out) device. The ring topology consists of two PLCs (one main device and a backup) to ensure reliable communication within the fieldbus network. This network redundancy is what makes this type of attack challenging because cutting the communication link of the main PLC will activate the backup until the attacker is able to close the loop again. The RIO allows communication between the PLC and the actuators and sensors by converting the digital signals required by the former and analog signals required by the latter before transmitting to the respective devices.

In the attack described in [67], the authors show that MitM attackers would be able insert themselves between the PLC and the actuators and sensors and consequently manipulate sensor reading and/or control commands sent to the actuators. This could lead to, among other things, tank overflows and the manipulation of the treatment process to contaminate the water and potentially damage tanks and pipes. The attackers could also launch a series of stealthy attacks which emulate the physical processes of the system in order to remain undetected by the built-in fault detection mechanisms of the control system. These types of attacks are not easy to carry out because an attacker first needs to have an intimate knowledge of how the system operates which he can gain by first launching passive attacks. These attacks are also more sophisticated than network traffic-based attacks (e.g., DoS attacks), which may not require prior knowledge of the system, but they are far more difficult to detect and have substantially more devastating consequences when successful [68].

### 6.2. Feature Selection

One of the most important considerations in machine learning is that of feature selection which will directly impact how well the algorithm will work in the application scenario. When considering feature selection for anomaly detection in critical water system infrastructure, it may be useful to first consider how fault detection mechanisms in these systems work. As mentioned in the previous section, data integrity anomalies are more difficult to classify and have more devastation consequences than network anomalies so that will be the focus of this discussion. These FD algorithms use the fact that ICS applications change the physical state of their application environments and these changes should thus obey the laws of physics [69]. For example, if a flow valve from a specific tank is opened, then the flow of water into/out of that tank should follow the laws of fluid dynamics. If the sensor readings and corresponding control commands do not result in physical changes that obey these laws then there is a fault in the system.

Knowledge of these laws above can be used to predict what the system state should be, given a set of time-series inputs, and if the actual value falls outside of a predetermined threshold it is flagged as a system fault. Using machine learning, designers of the algorithms do not need to have an in depth understanding of these laws because the detection algorithm would be able to learn the input/output relationship from historical data. A basic knowledge of these laws would however enable designers to choose the features that would produce the best results for their specific applications. Designers would also then be able to make adequate decision regarding the data pre-processing required before training commences.

### 6.3. Training Methods

As already mentioned in the previous sections, anomalies are rare obscure events in practice so labelled anomalous training data is hard to come by. This means that supervised learning as a classification problem would not yield good results because there might not be enough positive examples to train the algorithm. Based on the discussion in the previous section though, supervised learning can be used to learn the system behaviour and predict the system state given some time-series inputs. As with their fault detection counterparts, if the actual value is outside a predetermined error margin from the predicted value, it could then be flagged as an anomaly. In this case, labelled training data as it pertains to anomalous states is not required because the algorithm is learning how the system operated under normal condition and anything not adhering to that would then be classified as an anomaly. Anomalous examples are still required but not in the training process, they will rather be used to evaluate the proposed algorithms as will be discussed in the next section.

To use parametric schemes, the probability distribution of that data has to be known beforehand. This knowledge can then be used to fit the training set to the model by calculating statistical parameters such as mean and standard deviation. The probability that given time-series inputs fall within normal system parameters can then be calculated and if the value is smaller than some predetermined threshold it is classified as an anomaly. This does not require a labelled training set so it can also be viewed as an unsupervised learning problem. Their non-parametric counterparts do not make any assumptions about the underlying probability distribution of the data but are similar in the way an anomaly is classified. The lack of labelled anomalous training data can thus be solved by ignoring anomalous data in the training process and rather using the little that is available for evaluation purposes.

### 6.4. Algorithm Evaluation

The lack of labelled anomalous data in the application environment means that accuracy is not an adequate measure to evaluate whether or not an algorithm is performing well. This is because assuming that anomalous data makes up only 5% of a particular dataset, which is possible given how rare anomalies are, then an algorithm that always predicts that the data is normal with have an accuracy of 95%. This means that in anomaly detection schemes, the accuracy does not reveal how well the algorithm is classifying the data. In this scenario, the detection rates would be more adequate in order to determine the precision and recall of proposed schemes. Another adequate evaluation metric is the area under the receiver operating characteristic (ROC) curve which will be discussed later.

There is no universal approach to selecting appropriate features, training methods, algorithms and evaluation metrics. These are mostly application specific and what works in one scenario may not work as well in others. This section presented a general guideline of points to take into consideration when dealing with anomaly detection in critical water systems. The state of the art techniques presented in this paper will now be discussed to see how they measure up against each other and the practical consideration mentioned in this paper.

## 7. Observations and Recommendations

Two main considerations have to be taken into account when designing anomaly detection schemes for IWSN applications: (1) the resource constraints of the nodes and (2) the lack of training data [70]. The former requires the designer to consider the trade-offs between the detection accuracy and the computation requirements because as the detection accuracy increases then so too does the computational complexity. The latter requires the designer to be cognisant of the fact that supervised learning as a classification problem may not always be possible because the knowledge formulation process will not always be in their full control. The schemes discussed in this paper will now be compared taking this into consideration. It is important to note that this discussion is also applicable to cyber-physical systems as outlined in Section 5 and there will be many recurring themes here because many of the challenges are interchangeable. Table 3 shows a comparison of the anomaly detection schemes discussed in this paper.

### 7.1. Summary Comparison

As can be seen in Table 3, the parametric methods [34,35,36] all require prior knowledge of the data’s density distribution. This means that these methods aren’t flexible when considering the lack of control over the knowledge formulation process. All three schemes were found to have high detection accuracies and two of the schemes had a data recovery mechanism when an anomaly was detected. The majority of the discussed schemes did not consider data prediction, in fact only two of the non-parametric schemes did making it a total of four across all schemes. An important consideration is that all the schemes that included data prediction were specifically designed to detect data integrity anomalies which could have been the contributing factor. Going back to the parametric methods, the resource constraints of IWSNs were only considered in one of the methods but they all have theoretically low computational overheads thus making them feasible for these applications. IWSNs are however generally deployed in dynamic environments but these parametric methods are suitable for static environments where the data’s density distribution is not likely to change over time. The non-parametric methods are thus better suited for IWSNs because they are generally more robust but this comes at the cost of a higher computational overhead.

This paper focussed on non-parametric schemes that employed machine learning to detect anomalies. Of the KNN group of schemes discussed only [41] was specifically designed for resource constrained devices. The other two schemes discussed [42,43] did not consider the resource constraints and the former required large amounts of training data. For the non-parametric schemes, prior knowledge was indicated only if the scheme required a substantial amount of labelled training data. The scheme proposed in [41] achieves its efficiency through distributed computing and also uses unsupervised online learning thus eliminating the need for prior knowledge. In Table 3 it is however noted that the practical implications of the scheme were not considered. This is because the efficiency of the scheme was only considered theoretically and not backed up by any experimental data in the paper. All the non-parametric schemes recorded high detection accuracies with the exception of those proposed in [50,53]. The issue of high detection accuracies is however contentious and will be discussed in the next section.

Considering the SVM schemes [45,46,47] the practical implications were only considered in the one proposed in [45] which is also the only one that employs online learning techniques. However, like the one in [41] , the scheme was not specifically evaluated for a resource constrained environment as the practical feasibility was not backed up by any experimental data. Of the three ANN schemes [50,51,52] discussed two of them considered the practical implications and one of them required a substantial amount prior knowledge. Most of the remaining schemes required prior knowledge with the exception being the only GA scheme that considered the practical implications [54]. When considering all the non-parametric schemes just over a third of them required little to no prior knowledge and less than a third included substantial practical considerations. It is thus evident that conceptually there have been many proposed anomaly detection schemes for IWSNs that look very promising for this growing field. The practical feasibility has however been generally neglected in the literature and should start being given more attention going forward.

### 7.2. Discussion on Performance/Issues

As already mentioned at the beginning of the previous section the two main concerns when designing anomaly detection schemes for IWSNs are their resource constraints and lack training data. From the previous discussion it is clear that both of these primary concerns have been overlooked in the literature. Less than a third of the discussed non-parametric schemes considered the practical implications of the proposed schemes. Studies have however shown that many deployed IWSNs are error-prone and exhibit poor performances [71]. The resource constraints and distributed nature of IWSNs makes the design of these systems particularly challenging. Anomaly detection schemes run as monitoring agents on these already complex systems thus adding more complexity and further straining these already resource constrained systems. It is thus important for proposed schemes consider how they would function as part of these complex environments.

IWSN testbeds [72,73,74,75,76] are convenient tools to evaluate the feasibility of designed schemes in a practical environment. These testbeds are however costly and may not always be sufficient to evaluate complex machine learning algorithms but they provide a much clearer picture of how the schemes would fair in deployed full-scale systems than their simulation counterparts. None of the discussed schemes were evaluated using these testbeds, they instead made use of IWSN simulators such as TOSSIM [77] and NS-2 [78]. While these simulators do not accurately reflect the real world deployment environment, they are convenient tools in evaluating the feasibility and performance of proposed schemes and protocols. When considering the second important consideration, just over a third of the schemes did not require a substantial amount of prior knowledge. This is not ideal in IWSNs not only because labelled training data is not readily available in large quantities, but also because the algorithms have to be retrained if normal system behaviour changes. Considering the fact the IWSNs are deployed in dynamic environments and expected to last long periods of time, maintaining these schemes in a practical setting may not always be feasible.

Another important consideration is the evaluation metrics of the schemes. Given the vast range of IWSN applications, there is no universal benchmark that can be applied across the board. As discussed in the previous section, the detection accuracy in anomaly detection schemes is also not a good measure of how well the algorithm is actually working. This means that in practice these evaluation metrics should be evaluated based on the specific application scenario. While many of the papers do mention the accuracy and false positive rate, the false negative rate is rarely mentioned. In fact only three of the surveyed papers considered the impact of the false negative rate in their results. The precision and recall of the proposed schemes were rarely taken into consideration in the surveyed literature. Given the fact that anomalies are rare obscure events in practice, this factor should not be overlooked when considering how well the scheme classifies anomalies. The receiver operating characteristic (ROC) curve [79] is becoming an increasingly popular tool for evaluating machine learning algorithms. The curve is the true positive rate (*y*-axis) of the classifier plotted against the false positive rate (*x*-axis) obtained from the confusion matrix. Using these ratios instead of the standard numerical accuracies is a better performance measure because it includes all the data samples, even those that were erroneously missed by the classifier. In this way designers are able to thoroughly evaluate how well their classifiers are able to distinguish between normal and anomalous data. The area under the curve (AUC) has become a more effective performance measure than the standard numerical accuracy and many of the schemes found in the literature were evaluated using this approach. Given the fact that there is no universal benchmark however, there are also many schemes that don use this approach and even those that do use it vary appreciably in how they present their findings making a direct comparison of the schemes using this performance measure challenging.

### 7.3. Recommendations and Research Challenges

#### 7.3.1. Practical Evaluation

The resource constraints of IWSNs are one of their most critical limiting factors as discussed in the previous section. The literature survey revealed that very few of the proposed schemes’ feasibilities were adequately evaluated in this context. Future work should address this overlooked aspect in order for the field to evolve from being primarily concept-based, to one that is practically viable. Practical considerations are also more than just the resource constraints, in order to enhance their practical feasibility these schemes also have to include protocols that govern how they would work in a practical setup. Considerations of aspects such as the underlying network architecture, topology and protocols are paramount in determining a scheme’s viability in practice. Very few schemes found in the literature considered this and most were primarily evaluated using popular datasets such as those found on the University of California, Irvine (UCI) machine learning repository [80]. While this is adequate to evaluate the accuracy of the proposed schemes, it neglects the challenges inherent within the IWSN application environment.

#### 7.3.2. Complex Malicious Attack Protection

The vast range of IWSN applications means that they have different and often conflicting priorities. These applications thus have dedicated network policies and protocols to this end and anomaly detection schemes should form part of this while maintaining their detection generality. The majority of the schemes proposed in the literature were designed to detect generic anomalies. Considering the fact that anomaly detection schemes should also be able to detect anomalies that result from malicious attacks, which might be concealed, this might not always be adequate. For instance, attackers might be able to compromise several nodes and then launch a series of unobservable attacks [81] to systematically shut the system down or cause irreparable damage. Generic detection schemes may not be able to detect these kinds of attacks if not trained adequately which could be disastrous in critical infrastructure applications. This is especially true with schemes that employ reinforced learning as the new behaviour might be falsely learned as the new norm. Given the lack of training data, how to protect these schemes against various complex malicious attacks while maintaining their detection generality is going to be in interesting research topic going forward.

#### 7.3.3. Data Recovery

In a practical setup, sensor readings are inherently unreliable and inaccurate [46]. This means that nodes will frequently produce erroneous readings even though there is nothing wrong in the system. Anomaly detection schemes may falsely classify these random errors as anomalous (which they are) thus increasing the false alarm rate. Pairing anomaly detection algorithms with data recovery models or developing schemes with inbuilt predictive models would help alleviate this problem. Doing this helps in two ways, firstly the faulty readings can be replaced with the predicted values thus improving system performance, and secondly the overall accuracy of the detection scheme is improved. This often overlooked topic will be central in improving the feasibility of anomaly detection schemes in practice.

#### 7.3.4. Online Learning

The lack of labelled training data in IWSNs means that supervised learning as a classification problem will not be feasible for most applications. In cases where large training sets are available, a major drawback is that the algorithm would need to be retrained every time the normal system behaviour changes. Given the dynamic nature of IWSNs this is likely to happen often within the network lifetime. Online learning would thus be a feasible solution in the IWSN context. To achieve this detection generality however comes with its own challenges. For instance, the use of complex malicious attacks, as discussed previously, could result in the system learning new erroneous behaviour if the attacker remains undetected for a long enough period of time. This means that this detection generality could come at the cost of detection accuracy. Future work into this topic will have to consider the trade-off between the two and determine how much training data and which features are required to get the best possible outcome.

#### 7.3.5. Benchmarking

As previously stated, anomalies are rare obscure events in practice which makes detecting them very challenging. It has previously been stated that in the simulated scenario they are artificially created and often clearly defined. This will vary between different simulations and also will not necessarily translate well into practice. It thus becomes important for researchers to develop benchmarking tests that would enable other researchers to evaluate their schemes against an industry standard instead of developing their own tests, which could be misleading. These tests should take the practical implications of the application environment into consideration. This would give researchers a solid foundation on which to design their proposed even though the end results may be deployed in vastly different applications.

#### 7.3.6. Hybrid Schemes

Combining multiple detectors to create hybrid and ensemble schemes has the advantage of creating improved schemes that counteract each other’s limitations. This improvement in accuracy comes at the cost of a higher computational complexity which could render them infeasible for resource constrained IWSN applications. The authors in [58] however found that these schemes are in fact feasible in these applications depending on how they designed to function in this setup. Future work into this topic should look into innovative ways to combine multiple algorithms to produce schemes that perform better than the individual components but that also ensure that the resultant overhead would not render them impractical in a IWSN scenario.

#### 7.3.7. Critical Infrastructure Applications

Critical infrastructure applications employ cyber-physical systems to achieve their monitoring and control functionality. This improved functionality comes with numerous security challenges which need to be addressed because a compromised system could have disastrous real world consequences. Taking the previous discussions into consideration it is important to develop anomaly detection schemes that can fit into these complex environments in order to limit the damage of potential security breaches. In this context, it is not only important to detect the breach, but also to have recovery mechanisms in place to protect the underlying infrastructure once it has been detected. It is also paramount that these schemes be introduced while maintaining reliability and latency requirements of the system. These and the other requirements discussed in this paper have to be specifically considered for critical infrastructure applications. The implication of these schemes in this context should not be neglected in future work.

#### 7.3.8. Harsh Environments

IWSNs are sometimes deployed in extremely harsh environments such as underground mines [82] and intertidal habitats [83]. Deploying IWSNs in this scenario introduce several monitoring and communication challenges [84]. In addition to the inherent communication challenges such as high signal attenuation and poor link quality, the monitored environment is often dynamic and harsh which can inadvertently affect both the sensor readings and communication networks. In intertidal WSNs for example, packet delivery can be delayed by up to several days which can severely affect the operation of the deployed system [85]. From this discussion it is evident that distinguishing between anomalies and the inherent behaviour of IWSNs deployed these harsh environments is going to be an interesting research challenge for future work.

## 8. Conclusions

Abnormal changes within the environment where IWSNs are deployed can be detected using anomaly detection algorithms. This can be applied to many applications such as critical water systems. In these applications it is important to take the required prior knowledge and computation overhead into consideration when designing the schemes. Parametric methods are resource friendly but may not be applicable to many IWSN applications because they require the distribution function to be known in advance and also for it to remain static over the network lifetime. Machine learning techniques are more robust but this comes at the cost of a higher computational overhead. Conceptually there have been many proposed anomaly detection schemes for IWSNs that look very promising, which is a step in the right direction for this growing field. The practical feasibility has however been generally neglected in the literature and should start being given more attention going forward. Future work into the field should also consider aspects such as complex malicious attack protection, data recovery, online learning, and the development of robust hybrid and ensemble schemes. It is also important to develop schemes that can fit into complex cyber-physical environment given the critical nature of those applications. IWSNs are increasingly being used in many applications across a variety of different fields. Anomaly detection schemes are going to be an important protection mechanism as we move towards a more interconnected world.

## Figures and Tables

**Table 1:** Parametric vs. Non-parametric anomaly detection.

	Parametric	Non-Parametric
Prior-Knowledge?	Statistical distribution	Labelled training data
Environment?	Static	Dynamic
Detection speed?	fast	Moderate/slow
Detection generality?	No	Yes

**Table 2:** Comparison of anomaly detection training methods.

	Supervised	Semi-Supervised	Unsupervised
Prior-Knowledge?	Yes	Yes	No
Environment?	Static	Dynamic	Dynamic
Detection speed?	Fast	Fast/moderate	Moderate/slow
Detection generality?	No	Yes	Yes

**Table 3:** A comparison of the discussed anomaly detection schemes.

Scheme	Technique	Prior Knowledge	Complexity	Practical Consideration	Accuracy	Data Prediction	Anomaly	Drawback
Xie et al. [34]	Multivariate	Yes	Low	No	High	No	DOS	Dimensionality/PK
Magan-Carrion et al. [35]	Multivariate	Yes	Low	Yes	High	Yes	Data Loss/Modification	Affected by Routing/PK
Magan-Carrion et al. [36]	Multivariate	Yes	Low	No	High	Yes	Tampered Data	Traffic Imbalance/PK
Xie et al. [41]	kNN	No	Moderate	No	High	No	Generic	No Regression
Liu et al. [42]	kNN	Yes	High	No	High	No	Generic	Dimensionality
Zhu et al. [43]	kNN	No	High	No	High	No	Misbehaving Nodes	Complexity
Martins et al. [45]	SVM	No	High	No	High	No	Generic	Complexity
Salem et al. [46]	SVM	Yes	High	No	High	Yes	Data Integrity	Complexity/PK
Shilton et al. [47]	SVM	Yes	High	No	High	No	Generic	Complexity/PK
Cannady [50]	ANN	No	High	No	N/A	No	DOS	Complexity
Bosman et al. [51]	ANN	No	Moderate	Yes	High	No	Generic	Detection Bias
Yusuf et al. [52]	ANN	Yes	High	Yes	High	Yes	Data Integrity	Complexity/PK
Radhika et al. [53]	GA	Yes	High	No	Average	No	Misbehaving Nodes	Complexity/PK
Bankovic et al. [54]	GA	No	High	Yes	High	No	Misbehaving Nodes	Complexity
Rizwan et al. [55]	GA	Yes	High	No	High	No	Generic	Complexity/PK
Maleh et al. [56]	Hybrid	Yes	Moderate	Yes	High	No	DOS	PK
Ma et al. [57]	Hybrid	Yes	High	No	High	No	Generic	Complexity/PK

## Abbreviations

The following abbreviations are used in this manuscript:

IWSN	Industrial Wireless Sensor Networks
SCADA	Supervisory Control And Data Acquisition
IoT	Internet of Things
FD	Fault Detection
DoS	Denial of Service
kNN	K-Nearest Neighbours
LOF	Local Outlier Factor
COF	Connectivity-based Outlier Factor
ULOF	Uncertain Local Outlier Factors
WNaNG	Weighted Natural Neighbour Graph
SVM	Support Vector machines
SRM	Structural Risk Minimisation
RKHS	Reproducing Kernel Hilbert Space
RBF	Radial Basis Function
CS-SVM	Conic Segmentation SVM
ANN	Artificial Neural Networks
OS-ELM	Online Sequential Extreme Learning Machine
SLFN	Single Layer Feed-Forward Neural Networks
GS	Genetic Algorithms
NS-2	Network Simulator 2
NSA	Negative Selection Algorithm
AIS	Artificial Immune System
SC	Spectral Clustering
DNN	Deep Neural Networks
PLC	Programmable Logic Controller
SWaT	Secure Water Treatment
MitM	Man-in-the-Middle
ICS	Industrial Control Systems
RIO	Remote Input/Output
ROC	Receiver Operating Characteristic
AUC	Area Under the Curve
